# An Overview on Denial-of-Service Attacks in Control Systems: Attack Models and Security Analyses

**DOI:** 10.3390/e21020210

**Published:** 2019-02-22

**Authors:** Ahmet Cetinkaya, Hideaki Ishii, Tomohisa Hayakawa

**Affiliations:** 1 Department of Computer Science, Tokyo Institute of Technology, Yokohama 226-8502, Japan; 2 Department of Systems and Control Engineering, Tokyo Institute of Technology, Tokyo 152-8552, Japan

**Keywords:** networked control, cyber-security, denial-of-service, jamming attacks, probabilistic failure models, stability analysis, resilient control systems, multi-agent systems

## Abstract

In this paper, we provide an overview of recent research efforts on networked control systems under denial-of-service attacks. Our goal is to discuss the utility of different attack modeling and analysis techniques proposed in the literature for addressing feedback control, state estimation, and multi-agent consensus problems in the face of jamming attacks in wireless channels and malicious packet drops in multi-hop networks. We discuss several modeling approaches that are employed for capturing the uncertainty in denial-of-service attack strategies. We give an outlook on deterministic constraint-based modeling ideas, game-theoretic and optimization-based techniques and probabilistic modeling approaches. A special emphasis is placed on tail-probability based failure models, which have been recently used for describing jamming attacks that affect signal to interference-plus-noise ratios of wireless channels as well as transmission failures on multi-hop networks due to packet-dropping attacks and non-malicious issues. We explain the use of attack models in the security analysis of networked systems. In addition to the modeling and analysis problems, a discussion is provided also on the recent developments concerning the design of attack-resilient control and communication protocols.

## 1. Introduction

Many industrial control systems rely on information and communication technologies for their operation. In particular, wireless networks and the Internet are becoming key components of control systems, since they can be utilized in the transmission of measurement and control data to remote locations. As the Internet of Things is becoming more popular, the use of wireless technologies in control systems is expected to increase even more. These new developments are bringing efficiency to control systems, but they are also expected to introduce several vulnerabilities. A major concern is that cyber-attackers may be able to exploit the vulnerabilities in control systems that are utilized in power grids, transportation, water distribution, and many other services that are important for the society. To prevent financial losses and environmental damages that may be caused by disruption of those services, it is critical to assess and improve the security of existing control systems and develop new systems that are resilient against cyber attacks.

Various cyber-security issues of networked control systems and detection/mitigation approaches for those issues have been discussed in [1,2,3,4,5,6,7,8]. As pointed out in those works, attackers targeting vulnerable networked control systems may be able to change the contents of measurement and control packets. There may even be cases where attackers can inject false data into the system without being detected. These attacks require the attacker to be knowledgeable on the communication protocol as well as the system dynamics. On the other hand, attackers who have limited information on the control system may resort to denial-of-service (DoS) attacks that prevent delivery of control and measurement data packets. Networked control system under DoS attacks can face severe performance issues.

Our goal in this paper is to provide an overview of attack-modeling and security analysis approaches in recent works that explore networked control systems subject to DoS attacks. To this end, we look at the control, estimation, and consensus problems and discuss different DoS attack models utilized by researchers for addressing those problems. DoS attacks can take different forms in different network settings. In this paper, we focus on packet drop attacks by malicious nodes in multi-hop networks, and jamming attacks in wireless channels.

In multi-hop networks such as wireless ad hoc networks, remotely located nodes can transmit data packets to each other with the help of intermediate nodes that act as routers. It is typically assumed that all nodes obey the routing protocols in the network; however, in reality, a network can face packet drops by malicious nodes [9,10]. For instance, in *blackhole* DoS attacks, a malicious node first falsely introduces itself to other nodes as if it is part of the shortest path to a set of remote nodes. Then, many unsuspecting nodes in the network attempt to send all packets addressed to those remote nodes through the malicious node, which in fact drops all packets instead of forwarding them. Furthermore, in *grayhole* attacks, malicious nodes act normal and follow the standard routing protocols for certain periods of time to avoid being detected. The authors in [9,10,11,12,13] discussed both malicious and non-malicious packet dropping issues in ad hoc networks and presented several attack detection and mitigation approaches.

In addition to packet-dropping attacks, networked controls systems may also suffer from DoS attacks in the form of jamming of wireless transmissions. Specifically, a jamming attacker can prevent transmission of packets by emitting strong interference signals to a wireless channel [14,15]. It is mentioned in [15] that jamming devices can target various wireless technologies including GPS, mobile communications, and Wi-Fi. Jamming attacks can become a major concern for control systems, since they are easy to launch. The work in [16] illustrates that off-the-shelf hardware with wireless capabilities can be used for generating jamming attacks on wireless networks that use the popular IEEE 802.11 protocol. Jamming attacks can target both the physical layer and the medium access control (MAC) layer of the protocol. In the case of physical-layer attacks, the jamming attacker is not even required to follow the wireless protocol. By simply emitting strong interference signals, the jamming attacker can cause a decrease in the Signal to Interference plus Noise Ratio (SINR), which in turn prevents the receiver to detect transmitted packets [16]. In the case of MAC-layer attacks, both the packet sender and the jamming attacker operate on the same channel; the jamming attacker’s goal is to cause packet collisions [16]. Under jamming attacks, packets transmitted to the receiver may get corrupted and fail cyclic redundancy checks (CRC) used in the protocol. Note that, in wireless networks, jamming attacks and packet-dropping attacks by malicious nodes can also coexist.

Recently, DoS attacks have been investigated in the context of feedback control, state estimation, and multi-agent consensus problems. To tackle these problems, researchers have proposed several approaches for characterizing the occurrences of attacks. In particular, some of the works in the literature present models that take into account the energy constraints that an attacker may have. In another line of research, optimal attack strategies are investigated through game-theoretic frameworks and worst-case attack scenarios are studied. In this paper, we give an overview of both lines of research. In addition, we also discuss probabilistic modeling approaches that attempt to capture the effects of both malicious DoS attacks and non-malicious reasons of transmission failures. Such approaches are needed, as attacks may not be the only source of transmission failures in networks. Typically, networks may face non-malicious link errors, channel noise, and congestions caused by genuine network users [17,18]. In this paper, we discuss the utility of different modeling approaches in analyzing system security in control, estimation, and consensus problems. Furthermore, we present recent developments in the design of attack-resilient control and communication protocols.

We note that there are several review articles that discuss various aspects of denial-of-service attacks from the viewpoint of information technologies. In particular, denial-of-service attacks in sensor networks are investigated in [19]. A discussion of defense mechanisms against denial-of-service attacks is provided in [20]. Distributed denial-of-service attacks and potential approaches of mitigating their effects are explored in [21,22]. Additionally, an overview of a set of control-theoretical methods as well as techniques from information technologies to mitigate jamming attacks is presented in [23], and, furthermore, a survey of articles concerned with DoS and false data injection attacks in control systems for the smart grid is provided in [24]. Differently from previous works, we present an overview of DoS attacks in networked control systems with a special emphasis on probabilistic modeling and analysis approaches.

We organize the rest of our paper into five sections. In Section 2, we introduce control problems subject to DoS attacks and provide an overview of the literature that explore those problems. In Section 3, we discuss several deterministic, game-theoretical and optimization-based attack-modeling approaches. Moreover, in Section 4, we discuss recent developments in probabilistic approaches to modeling of networks that face denial-of-service attacks and also non-malicious issues. We then present a set of recently developed attack-resilient control and communication techniques in Section 5. Finally, we conclude the paper in Section 6.

We use fairly standard notation in the paper. Specifically, nonnegative and positive integers are, respectively, denoted by $N_{0}$ and N. Furthermore, the notation P[·] represents the probability on a probability space (Ω,F,P), and, moreover, E[·] represents the expectation. We use the notations ∨ and ∧ to, respectively, denote the “or” and “and” operations on binary numbers. Moreover, we denote the Lebesgue measure of a set S⊂R by |S|.

## 2. An Overview of Literature on Denial-of-Service in Control Systems

In this section, we present an overview of the recent literature that investigates DoS attacks in: (1) networked control; (2) state estimation, and (3) multi-agent consensus problems. We provide a general outlook on the problem settings and discuss a range of subproblems. These problem settings provide a basis for the more detailed discussions on attack models and security analysis techniques presented in Section 3 and Section 4.

### 2.1. Networked Control Problem

**Figure 1:**
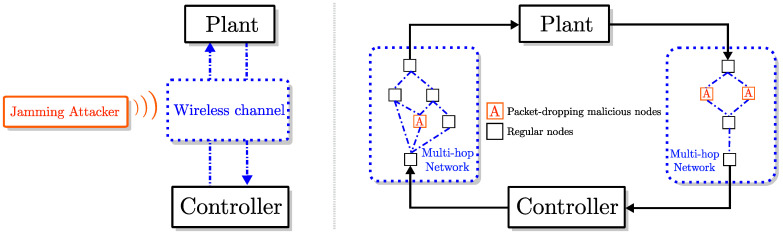
Operation of networked control system under denial-of-service attack: (**Left**) wireless networked control system facing jamming attacks; and (**Right**) multi-hop networked control system that faces packet-dropping attacks by malicious nodes in the networks.).

In the networked control problems depicted in Figure 1, the plant and the controller are remotely located entities that exchange data packets over a network that is subject to DoS attacks in the form of jamming and malicious packet-dropping. Such DoS attacks cause failures in the transmission of packets between the plant and the controller. Below, we discuss the networked control problem in both the discrete-time and the continuous-time settings.

#### 2.1.1. Discrete-Time Setting

We first look at the networked control problem of a discrete-time linear plant described by

(1) $$\begin{array}{cc} x(t+1) & =Ax\left(t\right)+Bu\left(t\right),\;x\left(0\right)=x_{0},\;t\in N_{0}, \end{array}$$

(2) $$\begin{array}{cc} y(t) & =Cx(t), \end{array}$$

where $x\left(t\right)\in R^{n}$, $u\left(t\right)\in R^{m}$, and $y\left(t\right)\in R^{h}$, respectively, denote the state, the input, and the output vectors of the plant. Moreover, $A\in R^{n\times n}$, $B\in R^{n\times m}$, and $C\in R^{h\times n}$, respectively, denote the state, the input, and the output matrices representing the dynamics.

In the state-feedback networked control problem, sensors located at the plant side measure the state x(·) and transmit it to the controller, which then computes a control input and transmits it back to the plant. In the output-feedback setting, the sensors measure and transmit the output y(·).

State-feedback networked control problem under DoS attacks is discussed in our previous works [25,26,27]. In those works, the system without control is considered to be unstable (i.e., *A* has eigenvalues that are outside the unit circle of the complex plane); moreover, the goal is to achieve stabilization of the zero solution x(t)≡0 by means of designing suitable control and communication rules. It is also assumed there that the network does not induce delay in transmissions, but packets may fail to be delivered due to attacks. In [28], we further generalized the setting so that delays can also be taken into account.

In the control frameworks in [25,26,27,28], the input u(t) that is applied to the plant is set to 0 when there is a failure in the transmission of the state measurement or the control input data. This is one of the most common approaches in the networked control literature that deals with transmission failures (e.g., Kellett et al. [29], Hespanha et al. [30], Gupta et al. [31], Okano and Ishii [32]). In the setup in [25,26,27,28], acknowledgement messages are not needed, since a packet exchange failure is known to the plant by the absence of an incoming control input. This in turn allows UDP-like communication protocols discussed in [33] to be used for the practical implementation of the networked operation.

In [26,27], we assumed that packet exchanges take place at every time step. On the other hand, in [25], we developed event-based communication (transmission) rules. In the event-based approach, packet exchanges are attempted between the plant and the controller at times $t_{0},t_{1},t_{2},\ldots \in N_{0}$ (with $t_{i}<t_{i+1}$). These times are decided based on certain event-triggering conditions. The triggering conditions proposed in [25] guarantee that the state stays within certain level sets in between consecutive transmission attempt times, and packet exchange attempts are triggered only before the state is predicted to leave a predefined level set. A more detailed explanation to this event-triggering setup is given in Section 5.1.1.

In the event-based setting in [25], the control input u(t) applied to the plant is given by

(3) $$\begin{array}{cc} u(t) & ≜\left(1-l(i)\right)Kx\left(\tau_{i}\right),\;t\in \left\{t_{i},\ldots ,t_{i+1}-1\right\}, \end{array}$$

where $K\in R^{m\times n}$ denotes the feedback gain matrix and ${\left\{l\left(i\right)\in \left\{0,1\right\}\right\}}_{i\in N_{0}}$ is a binary-valued process that is used for indicating success/failure of packet exchange attempts. Specifically, l(i)=0 indicates that the packet exchange attempt at time $t_{i}$ is successful, whereas l(i)=1 indicates that either the packet sent from the plant or the packet sent from the controller failed to be delivered at time $t_{i}$. If there are many packet exchange failures, the overall system can become unstable. This is because, when there is a failure, the system is governed by the unstable open-loop dynamics.

Notice that for the wireless networked control system depicted in Figure 1 (left), transmission failures happen due to *jamming attacks* [26]. For *multi-hop* networked control systems (Figure 1, right), packets are attempted to be transmitted over *multi-hop networks*, and packet exchange failures happen due to drops by one or more of the malicious nodes in the network [27].

It is also important to note that the strategy used by the attackers essentially determine which packet exchanges fail. Most of the works in the literature consider the problem where the strategy of the attacker is not known a priori. Typically, certain deterministic or probabilistic models are considered to characterize how frequent the attacks happen. In Section 3 and Section 4, we discuss such characterizations.

In the state-feedback control problem, Amin et al. [34] explored finite-horizon optimal control of a discrete-time linear plant under DoS attacks where timings of DoS can be random or arbitrary given that the total number of attacks in the horizon is bounded. Lai et al. [35] considered scenarios where a bound on the number of consecutive DoS attacks is known. Furthermore, in [26,36,37], we investigated the effects of jamming attacks by exploring physical wireless channel models based on Signal to Interference plus Noise Ratio (SINR).

In addition to the state-feedback control, the discrete-time output-feedback control problem has also been considered by taking into account the effects of DoS attacks. Specifically, Cetinkaya et al. [38], Wakaiki et al. [39] and Liu et al. [40] considered observer-based control ideas. In particular, Cetinkaya et al. [38] and Liu et al. [40] developed event-triggered controllers. The difficulty in the event-triggered output-feedback control problem is that the state information cannot be used for control purposes and for characterizing the event-triggering conditions. Observer-based quantized output-feedback control problem is investigated in [39], where a quantizer with dynamically varying ranges is utilized and sufficient convergence conditions are obtained. In addition to those results, optimal output-feedback control problem was considered by Zhang et al. [41] and Befekadu et al. [42] for systems with DoS attacks and noisy measurements. Furthermore, in [43], a game-theoretical approach is taken for an output-feedback networked control problem over multiple-channels that are subject to jamming attacks.

For discrete-time multi-hop networked control systems, there are several results (see, e.g., Cetinkaya et al. [27], Smarra et al. [44], D’Innocenzo et al. [45], D’Innocenzo et al. [46]). The multi-hop network characterization in our work [27] is based on a probabilistic approach that takes into account both non-malicious and malicious failures in the network (see Section 4.2). On the other hand, Smarra et al. [44], D’Innocenzo et al. [45] and D’Innocenzo et al. [46] utilized a different characterization where the information flow in the network for a given scheduling and routing protocol is characterized through difference equations. For this network setup, Smarra et al. [44] proposed methods for designing network weights as well as controller and observer gains by taking into account potential packet losses. Moreover, D’Innocenzo et al. [45] and D’Innocenzo et al. [46] proposed methods for detecting and isolating malicious nodes that intentionally delay packets or stop forwarding them. In addition, malicious nodes that inject false data can also be handled within the framework of [45,46].

#### 2.1.2. Continuous-Time Setting

In [47,48], researchers considered event-based remote control of a plant described by a linear continuous-time system

(4) $$\begin{array}{cc} \dot{x}\left(t\right) & =Ax\left(t\right)+Bu\left(t\right),\;x\left(0\right)=x_{0},\;t\geq 0, \end{array}$$

(5) $$\begin{array}{cc} y(t) & =Cx(t). \end{array}$$

The state of the plant is measured at times $t_{0},t_{1},t_{2},\ldots \in \left[0,\infty \right)$ and transmitted on the network to the controller. The control input applied at the plant side is kept constant between each successful transmission over the network. In particular, the input u(t) at the plant-side is given by

(6) $$\begin{array}{cc} u(t) & =Kx(t_{k(t)}), \end{array}$$

where k(t) denotes the index of the last successful transmission time. Notice that, in the control framework in [47,48], the control input at the plant side is not set to 0, when control data packet transmissions fail.

The framework developed in [47] allows resilient control update mechanisms. As we discuss further in Section 5.1.1, the intervals between consecutive transmission attempt times $t_{0},t_{1},t_{2},\ldots$ are designed to depend on the presence/absence of attacks. For instance, if the transmission attempt at time $t_{k}$ faces an attack, then the next transmission is attempted shortly afterwards, whereas, if the attempt at time $t_{k}$ is successful, the next attempt can be made after a longer duration. Similar resilient control setups are provided in the discrete-time setting in [25,38].

In [47,48], researchers provide a stability analysis approach for the closed-loop state-feedback control system in Equations (4) and (6) by utilizing bounds on the average duration and the frequency of DoS attacks. The characterization of attacks through average duration and frequency bounds is further discussed in Section 3.1.

The analysis approach in [47,48] does not require the strategy of the attacks to be known, and, moreover, the attacks can be time- or state-dependent. We also note that there are several works where periodic DoS attacks with unknown average duration and frequency are considered. In particular, Shisheh Foroush and Martínez [49] provided a detection-based control approach for periodic attacks, where the periodic strategy of the attacker can be learned so that transmission times are selected to avoid overlapping with the attack times.

In the case of *output-feedback* control problem, the utility of several different control frameworks under DoS attacks is investigated in [50,51,52]. As we further discuss in Section 5.1.1, Feng and Tesi [50] and Feng and Tesi [51] provided new architectures to limit the capabilities of attackers. Moreover, Lu and Yang [52] considered the case where multiple sensors make output measurements (*i*th sensor attempts to send the *i*th output measurement $y_{i}\left(t\right)=C_{i}x\left(t\right)$ over the *i*th channel).

In [53,54], the effect of DoS attacks on *nonlinear* systems is explored. Specifically, De Persis and Tesi [53] investigated the state-feedback control problem, and, moreover, Dolk et al. [54] explored dynamic event-based output-feedback controllers for stabilization. In [55], a linearization approach is developed for stabilizing nonlinear systems. There, it is mentioned that, when DoS attacks are sufficiently strong, the trajectories of the state may leave the linearization region, which may in turn cause instability due to the nonlinearity of the dynamics. For the case where the system dynamics involve unknown nonlinear functions, An and Yang [56] proposed adaptive controllers to guarantee boundedness of the state under DoS attacks.

Networked distributed control of a large-scale system is explored in [57]. There, the subsystems of the large-scale system utilize a shared network for the transmissions of their corresponding measurement and control input packets. To mitigate the effects of DoS, Feng et al. [57] proposed a transmission strategy that switches between event-based transmissions and a Round-robin protocol.

The performance of periodic and event-triggering networked control approaches under different types of jamming attacks was investigated in [58].

Additionally, frequency regulation problems in power networks under DoS attacks are considered in [59,60]. In both studies, the nodes in the power network are assumed to communicate over insecure communication channels that are subject to DoS attacks. In the problem formulation in [59], nonlinear dynamics are explored. Moreover, the researchers propose an event-based control approach in [60].

In [61], researchers considered networked control problem under DoS attacks and data-rate constraints, where state measurements are quantized through a dynamic quantization scheme.

We note that, among the different control approaches used for networked control systems under DoS attacks, event-triggered control (see, e.g., [25,38,48,54,58], and the references therein) appears to be the most commonly considered approach. We discuss the resiliency of event-triggering approaches against DoS attacks further in Section 5.1.1.

### 2.2. Networked State Estimation Problem

A remote state estimation problem over a network that is subject to DoS attacks is considered in [62]. There, the researchers explore a discrete-time linear plant given by

(7) $$\begin{array}{cc} x(t+1) & =Ax(t)+w(t), \end{array}$$

(8) $$\begin{array}{cc} y(t) & =Cx(t)+v(t), \end{array}$$

where $x\left(t\right)\in R^{n}$ and $y\left(t\right)\in R^{h}$, respectively, denote the state and output vectors; and $w\left(t\right)\in R^{n}$ and $v\left(t\right)\in R^{h}$, respectively, denote noises that are described by stochastic processes with zero-mean at each time instant *t*.

**Figure 2:**
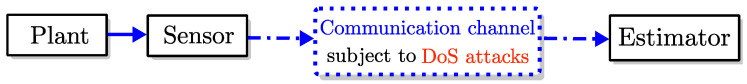
Operation of networked state estimation subject to DoS attacks.

The networked state estimation framework in [62] is shown in Figure 2. In particular, a sensor at the plant side is assumed to be able to compute a local estimate ${\hat{x}}_{S}\left(t\right)\in R^{n}$ based on output measurements y(t). For this purpose, a Kalman filter is utilized. At certain time instants, the estimate ${\hat{x}}_{S}\left(t\right)$ is sent over a communication channel to a receiving node representing a *remote estimator*.

As the receiving node may not have a direct access to the state estimate ${\hat{x}}_{S}\left(t\right)$ at all time instants, it keeps its own estimate $\hat{x}\left(t\right)$ of the state. If a new state estimate from the sensor is received, the receiving node sets its estimate value to the newly obtained value (i.e., $\hat{x}\left(t\right)={\hat{x}}_{S}\left(t\right)$). In the case where there is no transmission or the transmission fails due to DoS attacks, the receiving node cannot obtain any information. In that case, previously available state estimate is used together with the plant dynamics to obtain a predicted value by setting $\hat{x}\left(t\right)=A\hat{x}\left(t-1\right)$.

Li et al. [62] considered a finite horizon estimation problem, where T∈N denotes the horizon. In this problem, a performance indicator

(9) $$\begin{array}{cc} J_{\alpha }\left(T\right) & ≜\alpha \frac{1}{T}\sum_{t=1}^{T}E[∥x\left(t\right)-\hat{x}{\left(t\right)∥}^{2}]+\left(1-\alpha \right)E[∥x\left(T\right)-\hat{x}\left(T\right){∥}^{2}] \end{array}$$

with a scalar α∈[0,1] is used. By setting α=1, $J_{\alpha }\left(T\right)=J_{1}\left(T\right)$ represents the average estimation error variance, and by setting α=0, $J_{\alpha }\left(T\right)=J_{0}\left(T\right)$ corresponds to the final estimation error variance obtained at the end of the horizon.

In the problem formulation in [62], the sensor decides whether to transmit the data ${\hat{x}}_{S}\left(t\right)$ or not, and similarly the attacker decides whether to attack the channel or not at each time *t*. To identify the worst-case attack scenarios as well as the best transmission strategies, Li et al. [62] proposed game-theoretic characterizations, where the performance indicator $J_{\alpha }\left(T\right)$ is taken into account both by the sensor and the attacker. We explain these game-theoretic characterizations in a more detailed way in Section 3.2. In those characterizations, the number of transmissions by the sensor and the number of attacks by the attacker in a given horizon *T* are assumed to be constrained by certain scalars that are less than *T*. In [62], the optimal transmission and attack strategies are discussed for the case with constraints.

For scenarios where the sensor attempts transmission at each time step, closed-form optimal attack strategies that maximize $J_{1}\left(T\right)$ and $J_{0}\left(T\right)$ are obtained in [63,64]. The work in [63] also presents a variation of the problem of finding worst-case attack scenarios. In this variation, the attacker has additional constraints, which are proposed to explore strategies of an attacker that does not want to get detected by an intruder detection system.

The state estimation problem over wireless channels with SINR-based models have been discussed in [65,66]. Furthermore, Ding et al. [67] considered a network setup with multiple wireless channels. All three works explore game-theoretic and optimization-based analysis approaches. In particular, the sensor and the attacker are considered to be the players of a game. In the setting in [65,66], the players attempt to optimize not only the timing but also the signal and the interference power levels of transmissions and attacks. In the multi-channel estimation problem considered in [67], the sensor aims to optimally select the wireless channels that will be used to transmit packets, and, moreover, the attacker wants to optimally select the channels that will be blocked. For the state estimation problem with multiple sensors and multiple channels, the optimal strategies of the attacker are also discussed in [68].

We note that estimation problems under different attack types have also been studied. For instance, Guo et al. [69] investigated optimal false data injection attacks in state estimation problem. Furthermore, Guan and Ge [70] explored state estimation under jamming attacks as well as false data injections and developed a threshold-based detection method.

### 2.3. Multi-Agent Consensus Problem

The consensus problem for a multi-agent system under jamming attacks is considered in [71,72,73]. The multi-agent system in those works consists of *n* agents that possess scalar dynamics. In particular, the evolution of the *i*th agent’s state is described by

(10) $$\begin{array}{cc} {\dot{x}}^{i}\left(t\right) & =u^{i}\left(t\right),\;t\geq 0, \end{array}$$

where $x^{i}\left(t\right)\in R$ is the state and $u^{i}\left(t\right)\in R$ is the local control input for agent *i*.

Inter-agent communication topology of the multi-agent system is characterized with an undirected graph G=(V,E), where the nodes V={1,…,n} represent the agents and the edges E⊂V×V represent the communication links between agents.

The goal in [71,72,73] is to develop control and communication rules to guarantee practical consensus under jamming attacks. In practical consensus, agents states ${\left[\begin{array}{cccc} x^{1}\left(t\right) & x^{2}\left(t\right) & \cdots & x^{n}\left(t\right) \end{array}\right]}^{T}$ converge to a vector $x^{*}\in R^{n}$ that belongs to a consensus set $D_{\varepsilon }$

(11) $$D_{\varepsilon }≜\left\{x\in R^{n}:|\sum_{j\in N^{i}}\left(x^{j}-x^{i}\right)|<\varepsilon ,\;i\in V\right\},$$

where ε>0 represents a predetermined required level of closeness between agents and $N^{i}\subset V$ denotes the neighbors of agent *i*, that is, the set of agents that share a communication link with agent *i*.

**Figure 3:**
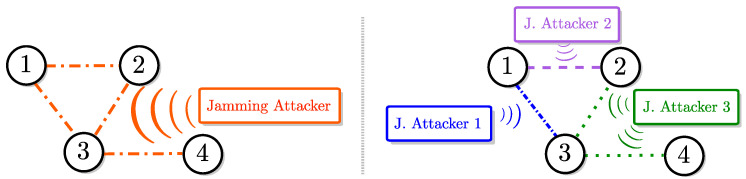
Multi-agent consensus in the presence of jamming attackers: (**Left**) single jamming attacker causes transmission failures on all inter-agent communication links; and (**Right**) multiple jamming attackers cause failures on different links).

For this problem formulation, Senejohnny et al. [71] and Kikuchi et al. [72] considered the attack setup shown on the left-hand side of Figure 3. In this setup, the jamming attacker can target all communication links at once. Specifically when there is an attack, none of the agents can communicate with each other. In the control approach in [71,72], each agent i∈V tries to communicate with its neighbors at certain times denoted by $t_{0}^{i},t_{1}^{i},\ldots$. Specifically, at time $t_{k}^{i}$, agent *i* sends a packet to its neighbors to request their states. In the case where there is no jamming attack at that time, all neighbors receive this packet and they respond by sending back their states. Those states are then used by agent *i* for constructing a ternary control input (i.e., $u^{i}\left(t\right)\in \left\{-1,0,1\right\}$) for achieving consensus.

For designing communication attempt times $t_{0}^{i},t_{1}^{i},\ldots$, Senejohnny et al. [71] used a self-triggering approach, where communication is attempted by each agent *i* only when a triggering condition holds. For achieving consensus, a restriction on the average duration and the average frequency on the attacks is imposed. These duration and frequency restrictions are discussed in Section 3.1, and they follow the attack characterization proposed by De Persis and Tesi [48]. It is shown in [72] that the restriction on the attack frequency can be removed when the agents utilize *randomization* in designing the communication attempt times. The utility of this randomization approach is further discussed in Section 5.2.1.

Recently, Senejohnny et al. [73] explored the multi-agent consensus problem under the attack setup shown in Figure 3 (right). In this setup, there are multiple jamming attackers that can target individual communication links.

We note that the consensus problem depicted in Figure 3 (left) is also explored for multi-agent systems with multi-dimensional dynamics in [74]. More recently, Nugraha et al. [75] considered a game-theoretical formulation of multi-agent systems under jamming attacks that can target individual links.

One of the key issues in studying the control, estimation, and consensus problems under denial-of-service attacks is that the attacker’s actions cannot be known a priori. To account for the uncertainty in the way the attacks may be generated, researchers have proposed several modeling approaches. These approaches can be classified into three groups: (1) deterministic approaches; (2) game-theoretic, optimization-based approaches; and (3) probabilistic approaches. In Section 3 and Section 4, we discuss these approaches in detail.

## 3. Deterministic and Game-Theoretical Approaches to Denial-of-Service Attack Modeling

In this section, we provide an overview of deterministic and game-theoretical approaches proposed in the literature for modeling denial-of-service attacks. We present several models and discuss their efficacy for addressing the networked control problems mentioned in Section 2.

### 3.1. Deterministic Attack Models with Average Duration and Frequency Constraints

In [47], the authors proposed a model that allows DoS attacks to happen in an arbitrary fashion as long as the total duration of attacks in a given time interval is upper-bounded by a deterministic function of the length of that interval.

**Figure 4:**
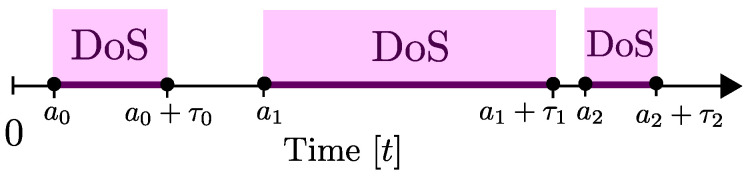
Sequence of DoS attack intervals. Transmission attempts that occur in any of the DoS attack intervals (represented with pink regions) fail.

In the continuous-time setting of this model, the timing of the attacks can be characterized as follows. First, two sequences ${\left\{a_{k}\geq 0\right\}}_{k\in N_{0}}$ and ${\left\{\tau_{k}\geq 0\right\}}_{k\in N_{0}}$ are used for denoting the starting times and durations of attack intervals. As we illustrate in Figure 4, the *k*th attack interval starts at time $a_{k}$ and lasts for $\tau_{k}$ units of time. It is assumed that $a_{k+1}>a_{k}+\tau_{k}$ so that different attack intervals are not overlapping.

This model fits well into scenarios with jamming, where the attacker may be emitting very strong jamming signals during the intervals $\left[a_{k},a_{k}+\tau_{k}\right]$. Note that, when a packet transmission time $t_{j}$ overlaps with any attack interval (i.e., $t_{j}\in \cup_{k\in N_{0}}\left[a_{k},a_{k}+\tau_{k}\right]$), then there will be a transmission failure at that time.

To model capabilities of the attacks, the notion of total attack duration is used. Specifically, for any time interval [τ,t]⊂[0,∞), the set A(τ,t)⊂[τ,t] is used for denoting the times that the network faces DoS attacks, i.e.,

(12) $$A\left(\tau ,t\right)≜\cup_{k\in N_{0}}\left[a_{k},a_{k}+\tau_{k}\right]\cap \left[\tau ,t\right],$$

and |A(τ,t)| is used for denoting the total duration of the attacks in the same interval.

Note that, in the case where DoS attacks cover the entire time span, we have |A(0,t)|=t for all t≥0. In such cases, communication on a network is not possible, and hence, control, estimation, and consensus goals cannot be achieved over such networks.

Typically, attackers may have constraints that prevent them from attacking at all times. For instance, in the case of jamming attacks, emitting powerful interference signals is costly and attackers with limited energy resources cannot conduct attacks at all time instants. Note also that the constraints may be imposed by the attacker who wants to avoid being detected. Strategic attackers may want to keep away from attacking continuously at all times to avoid detection.

The model in [47] takes into account such constraints by considering the following assumption.

**Assumption** **1.** *There exist scalars $\kappa_{D}\geq 0$ and $\rho_{D}\in \left[0,1\right)$ such that*

(13) $$\left|A\left(0,t\right)\right|\leq \kappa_{D}+\rho_{D}t,\;t\geq 0.$$

It follows from Equation (13) that ${lim sup}_{t\to \infty }\left|A\left(0,t\right)\right|/t\leq \rho_{D}$. This indicates that the scalar $\rho_{D}$ represents an upper-bound on the average ratio of attack durations in long intervals. If, for instance, $\rho_{D}=0.5$, then the total attack duration in the long run does not exceed 50% of the total time. In the case where the first attack interval starts at time $a_{0}=0$, Equation (13) implies a bound on the attack interval length $\tau_{0}$ as $\tau_{0}\leq \kappa_{D}/\left(1-\rho_{D}\right)$. Thus, the scalar $\kappa_{D}$ in Equation (13) can be selected for modeling initial capabilities of the attacker.

In [47,59], Assumption 1 is utilized in analyzing stability properties of networked control systems.

In [48], attackers with additional attack frequency constraints are considered. In particular, the following assumption characterizes attacks that have frequency constraints.

**Assumption** **2.** *There exist scalars $\kappa_{F}\geq 0$ and $\rho_{F}\in \left[0,1\right)$ such that*

(14) $$I\left(0,t\right)\leq \kappa_{F}+\rho_{F}t,\;t\geq 0,$$

*where $I\left(\tau ,t\right)\in N_{0}$ denotes the number of attack intervals in the time frame [τ,t].*

The scalar $\rho_{F}$ in Equation (14) provides an upper-bound on the frequency of attacks in the long run. To achieve stabilization of networked control systems with periodic transmissions, attack frequency needs to be bounded by a scalar $\rho_{F}$ small enough. To see this, first consider periodic transmission attempts at every Δ units of time. A strategic attacker who knows the transmission period Δ can concentrate his/her attacks to pinpoint the periodic transmission times with attacks that have very short durations (which satisfy Assumption 1). Thus, for such settings, all transmission attempts may fail if $\rho_{F}\geq \frac{1}{\Delta }$.

In [48], it is mentioned that Assumptions 1 and 2 do not suffice when one considers system dynamics with disturbance. This is because, under Assumptions 1 and 2, the attacker is allowed to attack continuously for arbitrarily long intervals as long as Equations (13) and (14) are satisfied. The attacker can achieve this by initially waiting for a long duration. This scenario is particularly dangerous for systems with disturbance, because even though the control packets may successfully be delivered in the initial attack-free period, the state never reaches zero due to disturbance. When the attack-free period ends, the attacker can attack continuously and cause the state to grow to very large values. To avoid such issues, De Persis and Tesi [48] proposed further restrictions of the average attack duration and the average attack frequency so that the maximum length of a continuous attack is bounded. These new restrictions are described by the inequalities

(15) $$\begin{array}{cc} \left|A(\tau ,t)\right| & \leq \kappa_{D}+\rho_{D}\left(t-\tau \right), \end{array}$$

(16) $$\begin{array}{cc} I(\tau ,t) & \leq \kappa_{F}+\rho_{F}\left(t-\tau \right), \end{array}$$

which are required to be satisfied for all τ≥0 and t≥τ. Notice that Equations (15) and (16) imply Equations (13) and (14), but not vice versa.

In [48,50,51], average duration and frequency conditions in Equations (15) and (16) are utilized for modeling attacks in a networked control problem.

It is shown in [48] that asymptotic stabilization with an event-triggered controller can be achieved under any attack strategy that satisfies Assumptions 1 and 2 with sufficiently small scalars $\rho_{D}$ and $\rho_{F}$. In particular, the sufficient condition given in [48] for asymptotic stability is

$$\begin{array}{cc} \Delta^{*}\rho_{F}+\rho_{D} & <\omega , \end{array}$$

where ω>0 is a scalar that depends on system dynamics, and $\Delta^{*}>0$ is scalar that upper-bounds the intervals between network transmission instants. It is shown in [48] that this condition guarantees input-to-state stability for systems with disturbance on the condition that $\rho_{D}$ and $\rho_{F}$ satisfy Equations (15) and (16) (in addition to satisfying Equations (13) and (14)). The authors of [50,51] considered periodic sampled-data controllers and showed that stability condition in this case can be made less conservative by using predictor and buffer mechanisms (see also Section 5.1.2). As noted in [48,50,51], $\kappa_{D}$ and $\kappa_{F}$ used in the inequalities in Equations (13) and (14) (or Equations (15) and (16)) affect bounds on state trajectories and the performance, but they do not affect the stability properties of linear systems. In the case of nonlinear systems, $\kappa_{D}$ and $\kappa_{F}$ play a role also in stability properties. In particular, $\kappa_{D}$ and $\kappa_{F}$ are utilized in [55] for determining the initial capabilities of the attacker and for obtaining conditions of stabilization with a controller that is designed through a linearization approach.

For the multi-agent consensus problem discussed in Section 2.3, [71,73] utilized the attack characterization with the conditions in Equations (15) and (16). It is observed in [72] that, with randomized transmissions, consensus in multi-agent systems can be achieved if the average attack duration is restricted as in Equation (15) regardless of the attack frequency.

In the discrete-time setting, a similar modeling approach can be utilized. The attack intervals can be defined similarly through sequences ${\left\{a_{k}\in N_{0}\right\}}_{k\in N_{0}}$ and ${\left\{\tau_{k}\in N_{0}\right\}}_{k\in N_{0}}$. In particular, the *k*th attack interval is given by $\left\{a_{k},a_{k}+1,\ldots ,a_{k}+\tau_{k}-1\right\}$. Again, it is assumed that $a_{k+1}>a_{k}+\tau_{k}$. Furthermore, average duration and frequency restrictions in Equations (13) and (14) as well as Equations (15) and (16) can be imposed in a similar way. For discrete-time networked control problems, Cetinkaya et al. [25] and Wakaiki et al. [39] considered average duration and average frequency restrictions in obtaining sufficient conditions of stabilization over networks under DoS attacks.

We note that there are other deterministic modeling approaches such as periodic DoS attacks considered in [49]. In particular, Shisheh Foroush and Martínez [49] modeled DoS attacks in a general way as a pulse width modulated (PWM) signal. In this model, the attacker repeats cycles of jamming and sleeping. Furthermore, in the special case of periodic jamming, each cycle consists of $T_{J}$ seconds of jamming that is followed by $T_{S}$ seconds of sleeping. Shisheh Foroush and Martínez [49] considered the setup where both $T_{J}$ and $T_{S}$ are unknown in the networked control problem. In addition, constraints on attacks were considered previously in a finite horizon problem by Amin et al. [34]. There, attacks can happen at arbitrary time instants given that the total number of attacks does not exceed a threshold for a given finite time horizon. As we further discuss in Section 3.2, such constraints have also been used in game-theoretic approaches.

### 3.2. Game-Theoretic and Optimization-Based Approaches for Attack Modeling

Game theory provides a natural framework for studying cyber security of control systems under DoS attacks and has been explored in many works (see, e.g., Li et al. [62], Li et al. [66], Ding et al. [67], Alpcan and Başar [76], Gupta et al. [77], Bhattacharya et al. [78]). Through a *game-theoretic* approach, researchers have obtained optimal DoS attack and defense schemes in certain problem settings. In what follows, we discuss some of the approaches in those works.

#### 3.2.1. Constraint-Based Models with Binary Actions

In several studies that deal with DoS attacks, the actions of the attacker and the defender are represented with binary variables corresponding to the choices between attacking and not attacking and similarly between defending and not defending.

For example, Li et al. [62] considered the finite-horizon state estimation problem discussed in Section 2.2, where binary variables $\lambda_{S}\left(t\right)\in \left\{0,1\right\}$ and $\lambda_{A}\left(t\right)\in \left\{0,1\right\}$ are used for indicating the decisions of the sensor and the DoS attacker, respectively. If, $\lambda_{S}\left(t\right)=1$, then the state estimate ${\hat{x}}_{S}\left(t\right)$ is attempted to be transmitted over the communication channel at time *t*. Moreover, the presence of an attack that causes a transmission failure at time *t* is represented by $\lambda_{A}\left(t\right)=1$.

Notice that, if the attacker and the defender do not have any constraints, it may be ideal for both players to act at every time instant (i.e., $\lambda_{S}\left(t\right)=\lambda_{A}\left(t\right)=1$ for all t∈N). In [62], researchers considered scenarios where both the attacker and the sensor have constraints represented by

(17) $$\begin{array}{cc} \sum_{t=1}^{T}\gamma_{S}\left(t\right) & \leq N_{S},\;\sum_{t=1}^{T}\lambda_{A}\left(t\right)\leq N_{A}, \end{array}$$

where T∈N is the time horizon of the estimation problem, and $N_{S}$ and $N_{A}$ are positive scalars that are strictly less than *T*. It is essential for the players to choose the best timing for their actions. To identify the worst-case attack scenarios as well as the best transmission strategies, Li et al. [62] proposed game-theoretic formulations, where a game is played by the sensor and the attacker. The goal of the defender is to minimize the cost in Equation (9) so as to minimize the estimation error, whereas the attacker wants to maximize Equation (9). For various constrained attack problems, closed-form optimal attack strategies $\lambda_{A}\left(1\right),\lambda_{A}\left(2\right),\ldots ,\lambda_{A}\left(T\right)$ that maximize $J_{1}\left(T\right)$ and $J_{0}\left(T\right)$ are obtained in [63,64].

A constraint on the total number of attacks in a given time horizon was considered also by Amin et al. [34] for a networked control problem under DoS attacks. Constraints similar to Equation (17) may characterize energy limitations of a jamming attacker. We note that, besides the formulation with constraints, there are also other formulations where the jamming energy is a part of the cost function of attacker (see, e.g., [76]). The effect of energy-related jamming costs was investigated by Zhu et al. [79], who considered a noncooperative game for analyzing the actions of a group of non-malicious nodes together with a malicious node that is capable of jamming and eavesdropping.

In addition to the energy constraints in Equation (17), the attacker may have additional constraints. In [63], some additional constraints are proposed to explore strategies of an attacker that does not want to get detected by an intruder detection system. It is discussed in [63] that a detection mechanism can check the number of transmission failures in the last $\tau_{D}\in N$ time steps and release an alarm if the failures go above a threshold $d_{D}\in N$. To avoid being detected by such a detection system, the attacker chooses a strategy that satisfies

(18) $$\begin{array}{cc} \sum_{t=k+1}^{k+\tau_{D}}\lambda_{A}\left(t\right) & \leq d_{D},\;k\in \left\{0,1,\ldots ,T-\tau_{D}\right\}. \end{array}$$

Notice that under Equation (18), the number of attacks in every consecutive $\tau_{D}$ time steps does not exceed $d_{D}$. An extension of this constraint to infinite-horizon problems resembles the constraints discussed in Section 3.1.

#### 3.2.2. SINR-Based Models

Recently, a few works considered Signal to Interference plus Noise Ratio (SINR) in modeling of the effect of jamming attacks to wireless communication channels (see, e.g., [66,80]). In those works, the probability of a packet transmission error depends on the Signal to Interference plus Noise Ratio (SINR), which is the ratio of the power of transmitted signal to the sum of the attacker’s interference power and the power of the channel noise. If the power of transmitted signal is small or interference and noise have large powers, then an error becomes highly likely.

In [66], researchers investigated the estimation problem over a wireless channel with an SINR-based model. In this model the sensor chooses a power level $p_{S}\left(t\right)\in \left[0,\infty \right)$ for transmitting the estimate ${\hat{x}}_{S}\left(t\right)$. In addition, the jamming attacker chooses an interference power $p_{A}\left(t\right)\in \left[0,\infty \right)$ at time *t*. The power values $p_{S}\left(t\right)$ and $p_{A}\left(t\right)$ affect the SINR given by $\frac{p_{S}\left(t\right)}{p_{A}\left(t\right)+\sigma^{2}}$, where $\sigma^{2}$ denotes the power of channel noise. The probability of a successful transmission at time *t* depends on SINR and is given by

$$\begin{array}{cc} 1 & -2Q\left(\sqrt{\alpha \frac{p_{S}\left(t\right)}{p_{A}\left(t\right)+\sigma^{2}}}\right), \end{array}$$

where $Q\left(x\right)≜\frac{1}{\sqrt{2\pi }}\int_{x}^{\infty }e^{-s^{2}/2}ds$, and α>0 is a parameter that depends on the wireless channel properties. Notice that successful transmission probability is affected by power levels $p_{S}\left(t\right)$ and $p_{A}\left(t\right)$ used by the sensor and the attacker.

In [66], optimal strategies of the sensor and the attacker are explored. In particular, first, a finite-horizon problem similar to the one in [62] is considered. The goal of the attacker is to find an optimal strategy $p_{A}\left(1\right),p_{A}\left(2\right),\ldots ,p_{A}\left(T\right)$ that maximizes a utility function while satisfying an energy constraint

$$\begin{array}{cc} \sum_{t=1}^{T}p_{A}\left(t\right) & =P_{A}, \end{array}$$

where $P_{A}>0$. In particular, the attacker’s utility is chosen as $TJ_{1}\left(T\right)$ with $J_{\alpha }\left(\cdot \right)$ given by Equation (9). The sensor’s utility function is considered to be the additive inverse of the utility of the attacker. Moreover, the energy constraint for the sensor is given by

$$\begin{array}{cc} \sum_{t=1}^{T}p_{S}\left(t\right) & =P_{S}, \end{array}$$

where $P_{S}>0$. A game-theoretic approach is taken in [66] to investigate optimal sensor and attack strategies. In [64], researchers considered a similar problem and provides an analysis on the optimal attack strategies $p_{A}\left(1\right),p_{A}\left(2\right),\ldots ,p_{A}\left(T\right)$ that yield the worst-case scenario in terms of the estimation performance. Optimal interference power levels and their effects were explored by Zhang et al. [80], who considered several settings of attack capabilities. In particular, Zhang et al. [80] considered both the case where the attacker can eavesdrop the acknowledgement messages and the case where the attacker has no knowledge of whether the attack is successful. In the case of infinite-horizon problems with an SINR-based formulation, Li et al. [66] showed that a *Q-learning* algorithm can be used by the sensor to obtain optimal strategies for selecting transmission powers.

A game with SINR-based formulation for wireless networked control systems was also considered by Yang et al. [81]. The players in the game are: (1) the user who decides the power of transmissions from the controller to the plant; and (2) the jamming attacker who decides the jamming interference power. In particular, Yang et al. [81] explored a *Stackelberg game*, where the user is the leader and acts first; the action of the user is then followed by that of the jamming attacker. In the problem setting in [81], the utilities of the user and the attacker depend directly on the SINR. They are expressed as $\frac{L\eta P}{\beta J+\sigma^{2}}$, where *L*, η, and β are fixed positive constants representing channel properties, $\sigma^{2}>0$ is the background channel noise, and P∈[0,∞) and J∈[0,∞), respectively, denote the transmission power of the user and interference power of the attacker. The cost of transmission and the cost of jamming for one unit of power are given respectively by the positive scalars *E* and *C*. Moreover, the utilities of the user and the jammer are, respectively, given by

$$\begin{array}{cc} V_{\mathrm{User}}\left(P,J\right) & ≜\frac{L\eta P}{\beta J+\sigma^{2}}-EP,\;V_{\mathrm{Jammer}}\left(P,J\right)≜-\frac{L\eta P}{\beta J+\sigma^{2}}-CJ. \end{array}$$

In this formulation, the optimal strategy of the jamming attacker for a given transmission power *P* is denoted by $J^{*}\left(P\right)$ and is obtained by finding J∈[0,∞) that maximizes $V_{\mathrm{Jammer}}\left(P,J\right).$ Then, based on the jamming attacker’s optimal strategy, the user’s best strategy is obtained in [81] through solving the problem

$$\begin{array}{c} \underset{P\in [0,\infty )}{\mathrm{maximize}}\;V_{\mathrm{User}}\left(P,J^{*}\left(P\right)\right). \end{array}$$

A Stackelberg game was also utilized by Liu [82] to investigate optimal attack and defense strategies in a multi-channel estimation problem. In this problem, a number of sensors acquire measurements of a plant and attempt to transmit these measurements to a remote receiver over insecure wireless channels. Each channel may be subject to a jamming attack and the transmission failure probabilities are characterized through an SINR formulation. In [82], two cases with incomplete information are considered: (i) the defender knows the probability of a possible attack and the total jamming power used on the wireless channels when there is an attack; and (ii) the defender knows the probabilities of possible attacks on each channel with the corresponding power levels.

In the context of multi-agent systems, Nugraha et al. [75] explored a game-theoretical formulation, where a game between a jamming attacker and a defender is considered by extending the infrastructure network modeling approach in [83]. In this game, the jamming attacker’s goal is to optimally choose communication links to attack, whereas the defender wants to recover those links. To this end, a new quantitative network connectivity notion is introduced in [75] to find optimal attack and defense strategies for scenarios where the attacker wants to decrease the connectivity by attacking certain communication links, whereas the defender wants to increase it by reestablishing the connection on the attacked links. In the problem, both players are assumed to have energy constraints that vary in time based on the previous actions of the players.

## 4. Probabilistic Approaches to Models of DoS Attacks and Non-Malicious Transmission Failures

In Section 3, we looked at deterministic and game-theoretic DoS attack models and analysis techniques. Our goal in this section is to discuss *probabilistic* approaches for modeling and analyzing the effects of DoS attacks. In particular, we focus on networks with wireless channels and multi-hop transmissions. For such networks, DoS attacks may not be the only source of failures, as transmissions may also fail due to non-malicious reasons [17,18]. For instance, wireless channels face unintentional interference, channel noise, and fading issues. Moreover, non-malicious issues such as link errors and congestion cause failures in multi-hop networks. In networked control systems, the transmission failures due to non-malicious issues are typically modeled through the use of stochastic processes such as Bernoulli processes [30,84] and Markov processes [31,32]. In [25], we observed that both non-malicious transmission failures and malicious DoS attacks as well as their combinations can be modeled through a *probabilistic* approach.

The probabilistic approach in [25] is developed upon tail-probability bounds for the binary-valued processes that describe the occurrences of transmission failures on a network. In what follows, we first explain this probabilistic approach and illustrate its generality by considering non-malicious failures and DoS attacks on a network. Then, in Section 4.1 and Section 4.2, we consider its utility in wireless channel and multi-hop network modeling.

Consider the binary-valued process ${\left\{l\left(i\right)\in \left\{0,1\right\}\right\}}_{i\in N_{0}}$ discussed in Section 2.1.1. This process is used for indicating the status of packet transmissions, where l(i)=1 represents a failure and l(i)=0 represents a successful transmission at time $t_{i}$. To describe the effects of certain non-malicious and malicious failure models in a unified manner, in [25,27], we investigated classes of binary-valued transmission failure indicator processes that describe networks where the number of failures in the long run is bounded in a probabilistic sense. First, for a given scalar ρ∈[0,1], consider the class $\Lambda_{\rho }$ of binary-valued processes given by

(19) $$\begin{array}{c} \Lambda_{\rho }≜\left\{l:l\left(i\right)\in \left\{0,1\right\},i\in N_{0};\sum_{t=1}^{\infty }P\left[\sum_{i=0}^{t-1}l\left(i\right)>\rho t\right]<\infty \right\}. \end{array}$$

Notice that the inequality $\sum_{t=1}^{\infty }P\left[\sum_{i=0}^{t-1}l\left(i\right)>\rho t\right]<\infty$ describes a condition on the tail probability $P[\frac{1}{t}\sum_{i=0}^{t-1}l\left(i\right)>\rho ]$ (i.e., the probability of the tail event where the ratio $\frac{1}{t}\sum_{i=0}^{t-1}l\left(i\right)$ of transmission failures exceeds ρ). Under this condition, the average number of failures is guaranteed to be bounded in the long run. In particular, a consequence of the Borel–Cantelli lemma [85] is that ${lim sup}_{t\to \infty }\frac{1}{t}\sum_{i=0}^{t-1}l\left(i\right)\leq \rho$, almost surely, for every $l\in \Lambda_{\rho }$. For the network control system discussed in Section 2.1.1, it is shown in [25] that the closed-loop system becomes stable if $l\in \Lambda_{\rho }$ for a sufficiently small ρ.

It was further noted by the authors of [25] that classes $\Lambda_{\rho }$ with different ρ values can be used to characterize binary-valued transmission failure indicators that are associated with networks that face: (1) non-malicious transmission failures; (2) malicious DoS attacks; and (3) combination of non-malicious and malicious issues.

In particular, to model non-malicious transmission failures, a time-inhomogeneous Markov chain ${\left\{l_{R}\left(i\right)\in \left\{0,1\right\}\right\}}_{i\in N_{0}}$ can be considered with initial distributions $\vartheta_{q}\in \left[0,1\right]$, q∈{0,1}, and time-varying transition probabilities $p_{q,r}:N_{0}\to \left[0,1\right]$, q,r∈{0,1}, satisfying

(20) $$\begin{array}{c} \begin{array}{c} P\left[l_{R}\left(0\right)=q\right]=\vartheta_{q},\\ P\left[l_{R}\left(i+1\right)=r|l_{R}\left(i\right)=q\right]=p_{q,r}\left(i\right),\;i\in N_{0}. \end{array} \end{array}$$

The following result shows that, if the probabilities $p_{0,1}\left(i\right)$ and $p_{1,1}\left(i\right)$ of transition towards the failure state 1 are bounded by a scalar $p_{1}\in \left(0,1\right)$, then $l_{R}$ belongs to the failure class $\Lambda_{\rho }$ for ρ values larger than $p_{1}$.

**Proposition** **3** ([25]). *Suppose $p_{0,1}\left(i\right)\leq p_{1}$ and $p_{1,1}\left(i\right)\leq p_{1}$, for $i\in N_{0}$, where $p_{1}\in \left(0,1\right)$. Then, $l_{R}\in \Lambda_{\rho }$ for any $\rho \in (p_{1},1]$.*

To model DoS attacks through the class $\Lambda_{\rho }$, consider a network with a single DoS attacker and let $l_{A}\left(i\right)\in \left\{0,1\right\}$ denote the possible actions of the attacker, where $l_{A}\left(i\right)=1$ represents an attack and $l_{A}\left(i\right)=0$ represents the absence of an attack. In certain scenarios, the DoS attacker has complete control of the network, and thus the values of l(i) can be solely decided by the actions of the attacker. In such scenarios, $l\left(i\right)=l_{A}\left(i\right)$. If $l_{A}\left(i\right)=1$ for all $i\in N_{0}$, then it means that all packets on the network fail to be transmitted. In the networked control problem, to achieve stabilization, some constraints on $l_{A}\left(\cdot \right)$ were considered by Cetinkaya et al. [25] through the following assumption.

**Assumption** **4.** *There exist scalars $\kappa_{A}\geq 0$ and $\rho_{A}\in \left[0,1\right)$ such that for every t∈N,*

(21) $$\sum_{i=0}^{t-1}l_{A}\left(i\right)\leq \kappa_{A}+\rho_{A}t,$$

*holds almost surely.*

Assumption 4 is similar to Assumption 1 in the sense that the inequality in Equation (21) places a restriction on the total number attacks by a certain ratio of the total number of transmission attempts. The scalar $\rho_{A}$ in this inequality is an upper-bound on the long-run average number of times the attacker attacks the network. As pointed out in [25], in the case of jamming, this scalar can be used for characterizing the energy use of the attacker and it is also related to the *jamming rate* mentioned in [86]. Notice that, in the context of jamming attacks, the formulation here corresponds to *reactive jamming*, where the attacker knows about the transmission times and directly attacks the network at those times.

It is mentioned in [14] that, in *reactive jamming*, the attacker monitors the channel and attacks it when a transmission is detected. This approach differs from *active jamming* attacks where the attacker’s goal is to simply prevent the use of a communication channel even if the channel is not currently used. Furthermore, as mentioned in [87], there are also situations where the attacker can decide to attack based on the content of packets. Specifically, in selective jamming attacks discussed in [87], a part of the content of the packet being transmitted can become available to the attacker who can then use this information to decide whether to send jamming signals to cause failure in the delivery of this packet. A similar malicious behavior is also seen in multi-hop networks where malicious nodes can drop certain packets based on their content [88].

Notice that the characterization in Assumption 4 provides a model based on a constraint on the total number of failures due to DoS attacks and it does not describe particular attack strategies. In this sense, it differs from the probabilistic attack models (Bernoulli- and Markov-modulated cases) considered in [34,42], where the attack strategies are described through probability distributions.

The following result from [25] shows that DoS attack indicator processes ${\left\{l_{A}\left(i\right)\in \left\{0,1\right\}\right\}}_{i\in N_{0}}$ satisfying Equation (21) belong to the class $\Lambda_{\rho }$ for $\rho \in (\rho_{A},1]$.

**Proposition** **5** ([25]). *Suppose ${\left\{l_{A}\left(i\right)\in \left\{0,1\right\}\right\}}_{i\in N_{0}}$ satisfies Assumption 4 with $\rho_{A}\in \left[0,1\right)$. Then, $l_{A}\in \Lambda_{\rho }$ for any $\rho \in (\rho_{A},1]$.*

Interestingly, the combination of non-malicious transmission failures and malicious DoS attacks can also be modeled with processes that belong to the class $\Lambda_{\rho }$ for certain values of ρ. To see this, first let the failure indicator process ${\left\{l\left(i\right)\in \left\{0,1\right\}\right\}}_{i\in N_{0}}$ be given by

(22) $$\begin{array}{cc} l(i) & =\left\{\begin{array}{cc} 1,\; & l_{R}\left(i\right)=1\;\;\mathrm{or}\;\;l_{A}\left(i\right)=1,\\ 0,\; & \mathrm{otherwise}, \end{array}\right.\;\;i\in N_{0}. \end{array}$$

The following result covers two cases: (1) non-malicious failures and DoS attacks occur independently, i.e., $l_{R}\left(\cdot \right)$ and $l_{A}\left(\cdot \right)$ are independent processesl and (2) the DoS attack strategy of the attacker may depend on non-malicious failures in the network, i.e., $l_{R}\left(\cdot \right)$ and $l_{A}\left(\cdot \right)$ are *not* independent.

**Proposition** **6** ([25]). *Consider a time-inhomogeneous Markov chain ${\left\{l_{R}\left(i\right)\in \left\{0,1\right\}\right\}}_{i\in N_{0}}$ with transition probabilities that satisfy $p_{q,0}\left(i\right)\leq p_{0}$ and $p_{q,1}\left(i\right)\leq p_{1}$, for $i\in N_{0}$, where $p_{0},p_{1}\in \left(0,1\right)$. Moreover, consider the process ${\left\{l_{A}\left(i\right)\in \left\{0,1\right\}\right\}}_{i\in N_{0}}$ satisfying Assumption 4 with $\rho_{A}\in \left[0,1\right)$. If $l_{R}\left(\cdot \right)$ and $l_{A}\left(\cdot \right)$ are independent processes and $p_{1}+p_{0}\rho_{A}<1$, then l(·) given by Equation (22) satisfies $l\in \Lambda_{\rho }$ for $\rho \in (p_{1}+p_{0}\rho_{A},1]$. If, on the other hand, $l_{R}\left(\cdot \right)$ and $l_{A}\left(\cdot \right)$ are not independent processes and $p_{1}+\rho_{A}<1,$ then l(·) given by Equation (22) satisfies $l\in \Lambda_{\rho }$ for $\rho \in (p_{1}+\rho_{A},1]$.*

The proof of this result utilizes a key lemma (see Lemma A.1 in [25]), where Markov’s inequality is used for obtaining Chernoff-type tail probability bounds for the term $P\left[\sum_{i=0}^{t-1}l\left(i\right)>\rho t\right]$. In the literature, Chernoff-type bounds are essential in obtaining concentration inequalities for sums of random variables (see Section 1.9 in [89], Chapter 27 in [90], and [91]).

**Figure 5:**
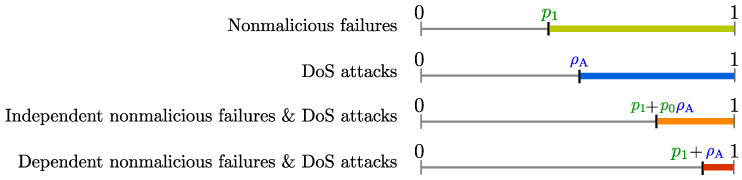
Ranges of ρ values for the class $\Lambda_{\rho }$. The ranges are different, when different transmission issues are considered in the network.

Notice that in the case where the DoS attack strategy of the attacker may depend on non-malicious failures in the network, $l\in \Lambda_{\rho }$ holds for larger values of ρ indicating that the average number of failures in the long run can be larger in that case. Ranges of ρ values associated with different transmission issues are illustrated in Figure 5.

### 4.1. SINR-Based Probabilistic Jamming Models

The authors showed in [26] that the probabilistic approach to characterization of DoS attacks proposed in [25] is also useful for modeling jamming attacks in wireless channels. In particular, transmission failures caused by an energy-constrained jamming attacker can be described by utilizing an SINR-based model similar to those in [66,80,82]. Specifically, Cetinkaya et al. [26] explored a networked control problem where the transmission power of the control input packets and the power of the channel noise are fixed constants. On the other hand, the interference power of the jamming attacker is allowed to depend on time and represented by the process ${\left\{v\left(i\right)\in \left[0,\infty \right)\right\}}_{i\in N_{0}}$. The likelihood of a transmission failure at a given time $t_{i}$ depends on the interference power v(i) of the attacker. If v(i) is large, then a transmission failure becomes more likely.

Our work [26] utilizes a Borel-measurable, nondecreasing function p:[0,∞)→[0,1] to describe the probability of failures. This function is used in the characterization of the transmission failure indicator l(i) as

(23) $$\begin{array}{cc} l(i) & ≜1[r(i)\leq p(v(i))], \end{array}$$

where r(i) is a random variable that is uniformly distributed in [0,1] for each $i\in N_{0}$. It is important to observe that Equation (23) implies

(24) $$\begin{array}{cc} P[l(i)=1|v(i)=\vartheta ] & =p(\vartheta ), \end{array}$$

that is, the conditional failure probability given that the jamming attacker sets the interference power to ϑ∈[0,∞) is represented by p(ϑ). It is assumed that r(0),r(1),… in Equation (23) are mutually independent so that failures occurring at different times are *conditionally independent* given the jamming interference powers at those times. In other words, for every $t_{1}<t_{2}<\cdots <t_{k}$, k∈N,

$$\begin{array}{cc} P[l\left(t_{1}\right) & =1,\cdots ,l\left(t_{k}\right)=1\left|v\left(t_{1}\right)=\vartheta_{1},\cdots ,v\left(t_{k}\right)=\vartheta_{k}\right]=\prod_{i=1}^{k}P\left[l\left(t_{i}\right)=1|v\left(t_{i}\right)=\vartheta_{i}\right]. \end{array}$$

In this setup, l(·) becomes a Bernoulli process with transmission failure probability P[l(t)=1]=p(ϑ), if v(·) is constant with v(i)=ϑ, $i\in N_{0}$.

Note that the function p(·) depends on SINR. For instance, if we consider the wireless channel setup in [66] (see Section 3.2.2), we set $p\left(v\right)=2Q\left(\sqrt{\alpha \frac{\zeta }{v+\sigma^{2}}}\right)$ where $Q\left(x\right)≜\frac{1}{\sqrt{2\pi }}\int_{x}^{\infty }e^{-\frac{s^{2}}{2}}ds$, α>0, and ζ∈(0,∞) and $\sigma^{2}\in \left(0,1\right)$ are, respectively, the transmission power and the power of the channel noise in the SINR $\frac{\zeta }{v+\sigma^{2}}$.

The energy constraints of the attacker are captured in [26] by means of introducing the following assumption, which places a bound on the average jamming interference power.

**Assumption** **7.** *There exist scalars $\kappa_{J}\geq 0$ and ${\bar{v}}_{J}\geq 0$ such that*

(25) $$\begin{array}{cc} P\left[\sum_{i=0}^{t-1}v\left(i\right)\leq \kappa_{J}+{\bar{v}}_{J}t\right] & =1,\;t\in N. \end{array}$$

In this assumption, ${\bar{v}}_{J}\geq 0$ characterizes the long-run upper-bound on the average interference power that can be utilized by the attacker. Moreover, $\kappa_{J}\geq 0$ determines the attacker’s initial capabilities. More specifically, ${lim sup}_{t\to \infty }\frac{1}{t}\sum_{i=0}^{t-1}v\left(i\right)\leq {\bar{v}}_{J}$, almost surely. Furthermore, $\frac{1}{t}\sum_{i=0}^{t-1}v\left(i\right)\leq \frac{\kappa_{J}}{t}+{\bar{v}}_{J}$ for the first *t* transmission attempts. In [37], we considered a more restricted version of Assumption 7 to study the joint effects of jamming interference and disturbance in networked control systems. The following result shows that, under Assumption 7, the failure indicator process l(·) defined in Equation (23) belongs to the class $\Lambda_{\rho }$ for certain values of ρ.

**Theorem** **8** ([26,36]). *Let $\hat{p}:\left[0,\infty \right)\to \left[0,1\right]$ be a continuous, nondecreasing, and concave function such that $\hat{p}\left(v\right)\geq p\left(v\right)$ for v∈[0,∞). Suppose that the transmission failure indicator l(·) is given by Equation (23), where the jamming interference power process ${\left\{v\left(i\right)\in \left[0,\infty \right)\right\}}_{i\in N_{0}}$ satisfies Assumption (7) with ${\bar{v}}_{J}\geq 0$. Then, $l\in \Lambda_{\rho }$ for $\rho \in (\hat{p}\left({\bar{v}}_{J}\right),1]$.*

**Figure 6:**
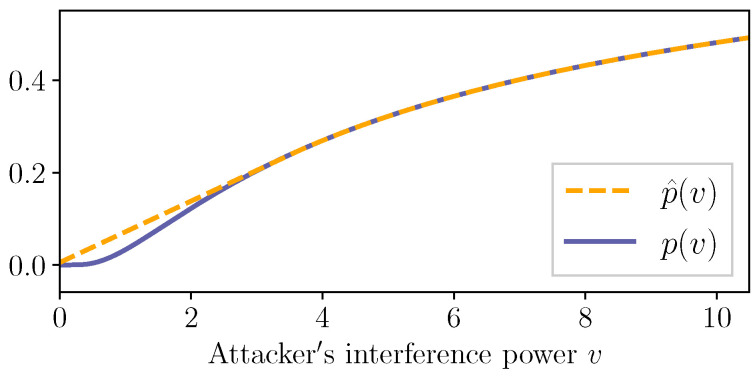
The transmission failure probability function *p* and a concave upper-bounding function $\hat{p}$ for an example wireless channel under jamming attacks.

Theorem 8 indicates that the probabilistic characterization through the class $\Lambda_{\rho }$ can also be utilized for wireless channels with SINR-based jamming models. Notice that the term $\hat{p}\left({\bar{v}}_{J}\right)$ provides the lower bound of the range of ρ for which $l\in \Lambda_{\rho }$ holds. Here, $\hat{p}$ is a concave function that upper-bounds the transmission failure probability function *p* (see Figure 6 for an example), and ${\bar{v}}_{J}$ is the upper bound of average jamming interference power. Theorem 8 is proved in [26] for the setup where the processes ${\left\{r\left(i\right)\in \left[0,1\right]\right\}}_{i\in N_{0}}$ and ${\left\{v\left(i\right)\in \left[0,\infty \right)\right\}}_{i\in N_{0}}$ are mutually independent. In the networked control problem discussed in Section 2.1.1, this assumption restricts the strategies of the attacker so that they are independent of the state and the control input information. Our recent work [36] shows that Theorem 8 also holds when ${\left\{r\left(i\right)\in \left[0,1\right]\right\}}_{i\in N_{0}}$ and ${\left\{v\left(i\right)\in \left[0,\infty \right)\right\}}_{i\in N_{0}}$ may depend on each other. The dependent case allows state-dependent attack strategies. In [36], a state-dependent attack strategy that maximizes the expected state norm in a rolling-horizon fashion is considered.

### 4.2. Multi-Hop Network Models

In addition to SINR-based jamming models, the probabilistic characterization through the class $\Lambda_{\rho }$ can be employed for modeling transmission failures in multi-hop networks. A multi-hop network (similar to those considered on the right diagram in Figure 1) can be represented by a directed acyclic graph G≜(V,E), where V is the set of nodes, and E⊂V×V is the set of edges. Note that V and E, respectively, represent the set of communication devices and the set of communication links. On a multi-hop network, data packets are transmitted over paths, which are sequences of non-repeating edges. Specifically, a *path* P from a node $v_{1}\in V$ to another node $v_{h}\in V$ is given as $P=\left(\left(v_{1},v_{2}\right),\left(v_{2},v_{3}\right),\ldots ,\left(v_{h-1},v_{h}\right)\right)$. Notice that there may be multiple paths between two nodes, and, moreover, those paths may be utilized in transmission of the same data.

**Figure 7:**
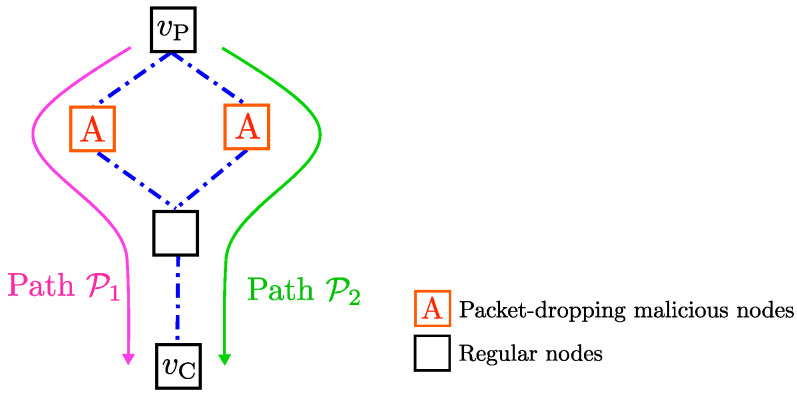
A multi-hop network between the plant ($v_{P}$) and the controller ($v_{C}$). State measurement packets on this network are transmitted over two paths $P_{1}$ and $P_{2}$, which are both subject to malicious packet-dropping attacks.

It is shown in our previous work [27] that the class $\Lambda_{\rho }$ can be utilized in modeling failures on individual links as well as paths and entire graphs. Specifically, in [27], a network G with source $v_{P}$ (corresponding to the plant) and sink $v_{C}$ (corresponding to the controller) ia considered (see Figure 7). The paths between nodes $v_{P}$ and $v_{C}$ are identified as $P_{1},P_{2},\ldots ,P_{\left|G\right|}$, where |G| denotes the total number of paths. Moreover, the individual links on the *i*th path are denoted as $P_{i,1},P_{i,2},\ldots ,P_{i,|P_{i}|}$, where $|P_{i}|$ is the number of links on path $P_{i}$. Transmission failures on those links can be represented by binary-valued failure indicator processes $l_{P_{i}}^{P_{i,j}}\left(\cdot \right)$. Similarly, overall failures on each path $P_{i}$ can be represented with an indicator process $l_{P_{i}}\left(\cdot \right)$, and, moreover, the failures of transmission between nodes $v_{P}$ and $v_{C}$ on network G can be represented with a binary-values indicator process $l_{G}$. Notice that $l_{G}\left(t\right)=l_{P_{1}}\left(t\right)\wedge l_{P_{2}}\left(t\right)\wedge \cdots \wedge l_{P_{\left|G\right|}}\left(t\right)$ and $l_{P_{i}}\left(t\right)=l_{P_{i}}^{P_{i,1}}\left(t\right)\vee l_{P_{i}}^{P_{i,2}}\left(t\right)\vee \cdots \vee l_{P_{i}}^{P_{i,|P_{i}|}}\left(t\right).$

The following result is obtained in [27] to show that the class $\Lambda_{\rho }$ can be used for characterizing the failure indicators for each individual link as well as the paths that include those links.

**Proposition** **9** ([27]). *Suppose $l_{P_{i}}^{P_{i,j}}\in \Lambda_{\rho_{P_{i}}^{P_{i,j}}}$, $j\in \{1,\ldots ,|P_{i}|\}$, where $\rho_{P_{i}}^{P_{i,j}}\in \left[0,1\right]$ and $j\in \{1,\ldots ,|P_{i}|\}$ satisfy $\sum_{j=1}^{|P_{i}|}\rho_{P_{i}}^{P_{i,j}}\leq 1$. Then, $l_{P_{i}}\in \Lambda_{\rho_{P_{i}}}$ with $\rho_{P_{i}}≜\sum_{j=1}^{|P_{i}|}\rho_{P_{i}}^{P_{i,j}}$.*

The next result shows that the overall failures on a network G can be characterized with the class $\Lambda_{\rho }$ where the ρ value is identified as the minimum of $\rho_{P_{i}}$ obtained for each path $P_{i}$ on the network G.

**Proposition** **10** ([27]). *Suppose $l_{P_{i}}\in \Lambda_{\rho_{P_{i}}}$ for each path $P_{i}$ where $\rho_{P_{i}}\in \left[0,1\right]$. Then, $l_{G}\in \Lambda_{\rho_{G}}$ with $\rho_{G}≜\min_{i\in \{1,\ldots ,|G|\}}\rho_{P_{i}}$.*

In [27], we present additional results, where more specific models for non-malicious failures and DoS attacks can be utilized in characterization of the network. It is important to note that in [27], we provide different methods to handle transmission failures due to data corruption and those due to packet dropping. In wireless multi-hop networks, data-corruption can be due to jamming attacks on the communication links, whereas packet-dropping issues are typically due to malicious nodes conducting blackhole or grayhole attacks (see [9]) and non-malicious routing protocols to avoid congestion.

## 5. An Overview of Attack-Resilient Control and Communication Techniques

In this section, we provide an overview on some of the recently developed control and communication techniques that aim to achieve resiliency against DoS attacks. These techniques rely on the modeling and analysis approaches discussed in previous sections.

### 5.1. Resilient Control Approaches

In what follows, we discuss event-triggered control schemes as well as predictor and buffer-based control frameworks.

#### 5.1.1. Event-Triggered Control

In the literature, one of the most common control strategies used against denial-of-service attacks is the *event-triggered* control. In the design and the analysis of event-triggered control schemes, the attack models presented in Section 3 and Section 4.1 are utilized.

In particular, for the continuous-time networked control problem discussed in Section 2.1.2, De Persis and Tesi [48] provided an event-triggered control mechanism that achieves asymptotic stabilization under any attack strategy that satisfies Assumptions 1 and 2. In their approach, a transmission of the state information is triggered when the error between the current state x(t) and the previously transmitted state exceeds a certain threshold.

It is mentioned in [48] that the event-triggering approach requires continuous monitoring of the state. Specifically, there is a need to continuously monitor the state x(t) to check if the triggering condition is satisfied or not. To avoid continuous monitoring, self-triggering approach is helpful. In the self-triggering approach proposed in [48], the predicted value of the state is used for calculating the next transmission time $t_{k+1}$. Notice that under DoS attacks, some of the transmissions may fail. The triggering approach in [48] takes into account the status of transmissions. If, for instance, the transmission attempt at time $t_{k}$ fails, then the next transmission is attempted shortly afterwards. If, on the other hand, the transmission at time $t_{k}$ is successful, the next transmission can be made after a longer duration. The parameters of the triggering schemes in [48] are designed to ensure that the overall control system is stable. Specifically, Lyapunov-function techniques are utilized for obtaining sufficient conditions concerning the event-triggering parameters that ensure global asymptotic stability under DoS attacks.

An interesting resilient event-triggered control strategy is proposed in [49]. In one of the scenarios considered in that work, jamming attacks on a communication channel happen periodically where the attacker repeats cycles of jamming and sleeping. For such attacks, control and transmission strategy in [49] can identify the transition times between jamming and sleeping states of the attack; thus, it allows selection of times where the communication is guaranteed to be successful. As a result, number of transmission attempts is further reduced.

**Figure 8:**
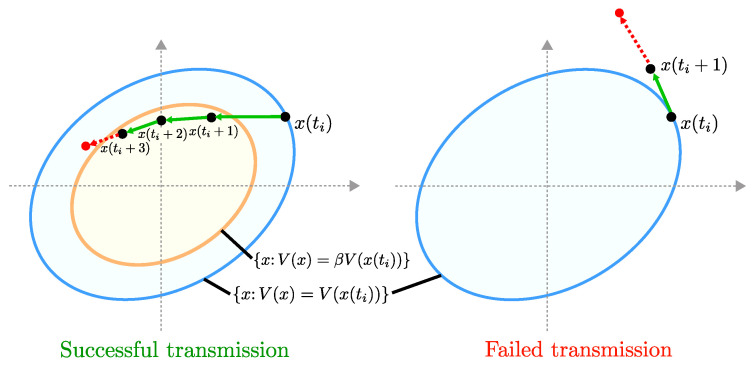
Illustration of the event-triggering approach in [25] for the cases of successful and failed transmissions at time $t_{i}$. A transmission is triggered at time $t_{i+1}$ when the state is predicted to make the move indicated with red dotted lines. In the case of successful transmissions (**Left**), the state is guaranteed to stay inside the level set $\left\{x\in R^{n}:V\left(x\right)=\beta V\left(x\left(t_{i}\right)\right)\right\}$ in between two event-triggering instants.

In addition, event-triggered control of a discrete-time networked control system (see Section 2.1.1) is explored in our previous works [25,38]. There, a Lyapunov-like function is utilized for determining the triggering time instants. Specifically, consider $V:R^{n}\to \left[0,\infty \right)$ given by $V\left(x\right)≜x^{T}Px$, where P>0. The network transmissions are attempted at times $t_{0}=0$, and $t_{i}$, i∈N, given by

(26) $$\begin{array}{cc} t_{i+1} & ≜\min\left\{t\in \left\{\tau_{i}+1,\tau_{i}+2,\ldots \right\}:t\geq t_{i}+\theta \;\;\;\mathrm{or}\;\;\;V\left(Ax\left(t\right)+Bu\left(t_{i}\right)\right)>\beta V\left(x\left(t_{i}\right)\right)\right\}, \end{array}$$

where θ∈N and β∈[0,1) are parameters of the event-triggered controller. The triggering condition $V\left(Ax\left(t\right)+Bu\left(t_{i}\right)\right)>\beta V\left(x\left(t_{i}\right)\right)$ guarantees that V(x(t)) stays within certain limits after a successful transmission at time $t_{i}$. As indicated in Theorem 11, the scalar β can be chosen so that when the transmission attempt at time $t_{i}$ is successful, then the closed-loop system shows stable behavior and the state goes inside a level set $\left\{x\in R^{n}:V\left(x\right)=\beta V\left(x\left(t_{i}\right)\right)\right\}$ at the next time instant. This is illustrated in Figure 8 (left). Furthermore, the next transmission event is triggered only when the state is predicted to leave this level set. For the example case shown Figure 8 (right), a transmission event is triggered at time $t_{i}+3$. If, on the other hand, the transmission at time $t_{i}$ is unsuccessful (see Figure 8, right), another transmission may be triggered in the next time instant if $V\left(Ax\left(t_{i}+1\right)+Bu\left(t_{i}\right)\right)>\beta V\left(x\left(t_{i}\right)\right)$. Notice that in this case the state trajectory indicates unstable behavior due to lack of control input in the system.

For the probabilistic network characterizations discussed in Section 4, sufficient stability conditions under the event-triggered control framework in [25] are provided in the following result.

**Theorem** **11** ([25]). *Consider the linear dynamical system in Equation (1). Suppose that the transmission failure indicator ${\left\{l\left(i\right)\in \left\{0,1\right\}\right\}}_{i\in N_{0}}$ satisfies $l\in \Lambda_{\rho }$ with scalar ρ∈[0,1]. If there exist a matrix $K\in R^{m\times n}$, a positive-definite matrix $P\in R^{n\times n}$, and scalars β∈(0,1),φ∈[1,∞) such that*

(27) $$\begin{array}{c} {\left(A+BK\right)}^{T}P\left(A+BK\right)-\beta P\leq 0, \end{array}$$

(28) $$\begin{array}{c} A^{T}PA-\varphi P\leq 0, \end{array}$$

(29) $$\begin{array}{c} (1-\rho )\ln\beta +\rho \ln\varphi <0, \end{array}$$

*then the event-triggered control law in Equations (3) and (26) guarantees almost sure asymptotic stability of the zero solution x(t)≡0 of the closed-loop system dynamics.*

The scalars β∈(0,1) and φ∈[1,∞) in the conditions in Equations (27) and (28) of Theorem 11 characterize upper bounds on the growth of the Lyapunov-like function $V\left(x\right)=x^{T}Px$, when the system evolves with the closed-loop dynamics (corresponding to a successful transmission) and open-loop dynamics (corresponding to a transmission failure). Notice that since β<1 and lnβ<0, if ρ is sufficiently small, then Equation (29) holds indicating stability. Based on the conditions of Theorem 11, our work [25] also provides a method for designing the scalar β and the positive-definite matrix *P* that are utilized in the event-triggering condition in Equation (26). The proof of Theorem 11 is based on a technique similar to that used for obtaining upper bounds of *top Lyapunov exponents* (see [92,93]) of stochastic dynamical systems. In [28], we provided a less conservative analysis approach based on a lifting technique. There, the stability of the system is checked by solving a linear programming problem.

#### 5.1.2. Control Frameworks with Predictors and Buffers

To mitigate the effects of DoS attacks in an output-feedback networked control problem, Feng and Tesi [51] introduced a controller with a predictor and an impulsive observer. In that work, the output y(t) of the system in Equations (4) and (5) is measured periodically and transmitted over a network that faces DoS attacks. Moreover, the control input packets are assumed to be transmitted over a secure network.

In [51], the system dynamics involve disturbance, and the transmitted output measurements can be noisy. The observer and the predictor at the controller side are designed in a way to ensure that accurate state estimates are obtained at the controller side after a certain number of successful transmissions. In particular, in the case without disturbance and noise, the state estimate at the controller matches the actual state if μ number of consecutive output measurements can be received at the controller side, where μ∈{1,…,n} is the observability index of the pair $\left(e^{A\Delta },C\right)$ with Δ denoting the output measurement/transmission period. When μ consecutive measurements are not available, the knowledge of the system dynamics is utilized in the predictor to predict future state values.

For this framework, it is shown in [51] that the closed-loop system stability can be preserved under any attack strategy satisfying the constraints in Equations (15) and (16), if the scalars $\rho_{D},\rho_{F}$ in those constraints also satisfy $\rho_{D}+\Delta \rho_{F}<1-\left(\mu -1\right)\Delta \rho_{F}$. It is important to note that this condition is independent of the choice of control gain. Note also that if instead of the output measurements, the state measurements are sent to the controller (i.e., μ=1), then the condition takes the form

(30) $$\begin{array}{cc} \rho_{D}+\Delta \rho_{F} & <1. \end{array}$$

This inequality shows that the predictor based approach guarantees stability regardless of the dynamics of the open-loop and the closed-loop systems.

When the control input packets are transmitted over channels that also face DoS, the right-hand side of the condition in Equation (30) is replaced by a term that depends on system dynamics. The work in [50] considers such scenarios, where DoS attacks affect the delivery of both control and measurement packets. There, a buffer is utilized at the plant side. The controller at each time sends the current control input together with future control inputs. When there is no DoS attack, the transmitted control input packets are placed in the buffer so that they can be utilized later when the attacker becomes active and blocks transmissions. It is shown in [50] that for sufficiently large buffer sizes, the closed-loop stability can be guaranteed for attacks that satisfy the condition in Equation (30).

### 5.2. Resilient Communication Techniques

Besides the event-based communication approaches discussed in Section 5.1.1, there are a few other communication techniques specifically developed for achieving resiliency in multi-agent consensus as well as in networked state estimation problems. In what follows, we provide an overview of those protocols.

#### 5.2.1. Self-Triggered and Randomized Communication Techniques in Multi-Agent Consensus

In [71,73], the authors explored the multi-agent consensus problem in Section 2.3, where the network is subject to jamming attacks. In those works, one of the deterministic attack models discussed in Section 3.1 is utilized, and a self-triggered communication rule is developed. The utility of the self-triggered approach is that with self-triggering, inter-agent communications are asynchronous and agents are not required to possess synchronized clocks. Furthermore, the self-triggered communication technique provides resiliency against a large class of attack strategies.

In the self-triggered communication technique, the (k+1)th communication attempt time $t_{k+1}^{i}$ for the agent *i* is determined based on the information available to agent *i* at time $t_{k}^{i}$. If the *i* agent knows its neighbors’ values at time $t_{k}^{i}$, then it uses this information in designing the next communication attempt time $t_{k+1}^{i}$. If, on the other hand, no information is available at $t_{k}^{i}$ (due to jamming attacks), the *i*th agent sets the waiting time until $t_{k+1}^{i}$ to be a fixed duration.

The minimum interval between consecutive communication attempts of all agents is given in [71] by $\Delta^{*}>0$. It is noted in [71] that consensus can be achieved under any attack strategy subject to the constraints in Equations (15) and (16) with scalars $\rho_{D}$ and $\rho_{F}$ that satisfy

(31) $$\begin{array}{cc} \rho_{D}+\Delta^{*}\rho_{F} & <1. \end{array}$$

The inequality in Equation (31) guarantees that the average duration and the average frequency of attacks are sufficiently small. Note that, if the attacker can attack at a frequency that is larger than the frequency of communication attempts (i.e., $\rho_{F}>\frac{1}{\Delta^{*}}$), then the attacker may be able to block all attempts of communication by the agents. The reason is that the attacker may actually be knowledgeable on the self-triggered mechanism used by the agents for deciding the times $t_{k}^{i}$. This would allow the attacker to attack the network directly at those instants for very short durations. In such cases, the attacker can preserve energy and satisfy Equation (15) for arbitrarily small $\rho_{D}$.

**Figure 9:**
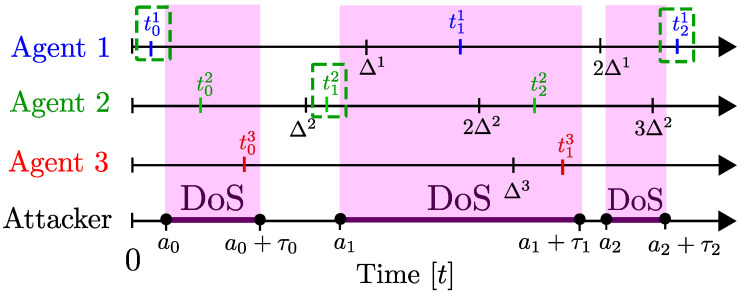
Illustration of the randomized communication protocol, where each agent attempts communicating with its neighbors at random time instants. At time instants denoted inside rectangles with green dashed borders, the communication attempts can avoid DoS attacks.

To achieve resilient consensus against attacks with high frequency, [72] proposed a randomized communication strategy. The key idea in the randomized setting is that the agents pick their communication times randomly (see Figure 9). In particular, each agent *i*, first picks a deterministic interval length $\Delta^{i}>0$. Then, for each $k\in N_{0}$, the communication attempt time $t_{k}^{i}$ is selected as a random variable that is uniformly distributed in the interval $\left[k\Delta^{i},\left(k+1\right)\Delta^{i}\right)$.

Randomized transmissions prevent the attacker to know about future communication attempt times of agents. It is shown in [72] that consensus in the randomized communication setting is achieved if the attack constraint in Equation (15) holds with $\rho_{D}$ that satisfy

(32) $$\begin{array}{cc} \rho_{D} & <1. \end{array}$$

In other words, the randomized communication approach guarantees consensus under any attack strategy that is only constrained in its average duration as in Equation (15) regardless of its frequency.

#### 5.2.2. Fake Acknowledgement Messages in State Estimation

In a networked state estimation problem, an interesting communication technique was considered by Ding et al. [65]. In the problem formulation, Ding et al. [65] considered an estimator that attempts to transmit state estimates to a remote receiver over an insecure wireless channel. The decision to attempt transmission or not is made by the estimator in a stochastic fashion by setting a probability value $\theta_{S}\left(t\right)\in \left[0,1\right]$ such that a transmission attempt at time *t* is made with probability $\theta_{S}\left(t\right)$.

When a transmission attempt is made at time *t*, it is not guaranteed that it will be successful due to the jamming attacks on the channel. The likelihood of a transmission failure is determined based on the power level of the jamming interference signal emitted to the wireless channel by the attacker at that time instant.

The goal in [65] is to minimize the time-averaged variance of the estimation error. To this end, Ding et al. [65] proposed the idea of generating a *fake acknowledgement message sequence* ${\left\{\phi \left(t\right)\in \left\{0,1\right\}\right\}}_{t\in N}$. Instead of sending real acknowledgements, the receiving node follows the fake acknowledgement sequence and sends back to the sensor acknowledgement messages that are possibly misleading for the attacker. For instance, if ϕ(t)=1, the receiving node sends a positive acknowledgement indicating that a packet is successfully received, even if no packet is received at time *t*. Under certain assumptions on the jamming interference powers, the work [65] derives the optimal transmission attempt probabilities ${\left\{\theta_{S}\left(t\right)\right\}}_{t\in N}$ as well as the optimal fake acknowledgement message sequence ${\left\{\phi \left(t\right)\right\}}_{t\in N}$ that minimizes the time-averaged variance of the estimation error. It is shown that fake acknowledgements improve the estimation performance.

#### 5.2.3. Design of Routing Protocols to Ensure Security Against DoS

As discussed in [94,95], denial-of-service can be a big problem in the delivery of packets in multi-hop networks. To increase the resilience of multi-hop networks against attacks, several works proposed routing protocols. In particular, for the case of mobile ad hoc networks (MANET), where the network topology is time-varying, the authors of [12,96,97] developed routing protocols that can avoid certain attacks. In the protocol developed in [12], each node keeps a list of weights for its communication links, and these weights are utilized for detecting faulty/attacked links and discovering reliable communication paths. Sanzgiri et al. [96] showed that authentication based techniques with cryptographic certificates can be utilized for detection of malicious and faulty nodes. Moreover, Bianchi et al. [97] proposed a routing mechanism that is based on identifying potentially cooperating malicious nodes that launch blackhole attacks in a multi-hop network. In the context of multi-hop networked control, the detection of malicious nodes and their isolation is explored in [45,46].

We note that, in addition to the attack-resilient control and communication techniques discussed above, there are also a few works that focus on denial-of-service attack detection in control systems. In particular, the authors of [63,98] discussed threshold-based attack detection mechanisms, and the analysis of transmission failure patterns to distinguish strategic attacks and non-malicious failures was explored by Cetinkaya et al. [26]. In addition, Ali et al. [99] recently considered methods from information technologies for detecting and mitigating distributed DoS attacks against networked control systems.

## 6. Conclusions

In this paper, we present an overview of the literature on denial-of-service attacks in control systems. In particular, we present a list of problems considered by researchers in the fields of networked control, networked state estimation, and multi-agent consensus. We provide a discussion on deterministic and game-theoretic approaches to modeling attacks on networks. We then focus on a probabilistic approach for characterizing the attacks in wireless channels as well as multi-hop networks. The notion of constraints can be considered as a common theme that connects the different modeling approaches. In particular, the cost of attacks and the energy available to the attacker play a role in most of the derived models. We discuss the utility of these models for analyzing the security of existing systems as well as for developing new attack-resilient control and communication techniques.

It appears that the detection problem for DoS attacks can be an interesting future research topic in control-system studies. Thus far, this problem has been explored in only a few works, and we think that some of the techniques from information technologies can be useful in investigation of DoS attack detection and mitigation problems in control systems if the dynamical properties of the system can also be taken into account.

As more and more control systems are expected to incorporate wireless technologies, it seems that the risk of DoS will also increase, making cyber-security of control systems against DoS an even more important research field.
